# Update on canine anorchia: A review

**DOI:** 10.1002/vms3.1033

**Published:** 2023-01-03

**Authors:** Vincenzo Cicirelli, Matteo Burgio, Daniela Mrenoshki, Sandor Cseh, Giulio Aiudi, Giovanni Michele Lacalandra

**Affiliations:** ^1^ Dipartimento di Medicina Veterinaria Università degli Studi di Bari Aldi Moro Bari Italy; ^2^ University of Veterinary Medicine Budapest Hungary

**Keywords:** anorchia, cryptorchidism, dog penis, urogenital tract

## Abstract

Abnormalities of the external genitals are an important issue in dog breeding because of the unfavourable qualities and characteristics of breeds, resulting in consistent economic losses. Despite their significance, little scientific attention has been given to these problems. Although there are several reviews on cryptorchidism in dogs, none have described anorchia. Testicular agenesis is a rare reproductive disorder with a congenital origin. Moreover, no author has described the diagnostic procedure for making a definitive diagnosis of anorchia in dogs. It is important to have a well‐structured diagnostic scheme to help practical veterinarians make a confirmatory diagnosis. This review article aims to provide an update on canine anorchia diagnosis based on the poor research studies published in recent years. We have also contributed to the pathogenesis of this disorder using human medicine studies. Finally, the review includes therapeutic hypotheses that can be expanded in future studies.

## Graphical abstract

The diagnostic procedure for anorchia in dog: A review

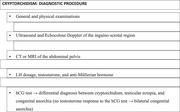

## 1 CONTEST

In recent years, scientific research studies have been encouraged by increasing knowledge among dog owners about sexual disorders, increasing awareness on the part of breeders to establish a genetic pool without defects, and by a greater understanding among veterinarians of this scenario. In fact, dog reproductive problems cause substantial financial losses (Cicirelli et al., 2021). In the literature, there are two reviews on cryptorchidism in dogs, one published in 1991 (Romagnoli, 1991), and a more recently published review with several updates (Khan et al., 2018). In contrast, anorchia, which is the complete absence of testicular tissue in dogs, has never been investigated. This review article aims to provide an update on canine anorchia based on the poor research studies published in recent years. Significant progress has been made in the investigation of sexual disorders in domestic animals in recent decades; however, the mechanisms underlying this problem in dogs are still not clear (Lyle, 2007). These studies treated cryptorchidism in general and discussed the genetic mechanisms underlying normal testicular descent in dogs and the aberrations that lead to failure of the descent of the testes (Adam et al., 2012). A few studies have focused on investigating newer methods for the diagnosis of cryptorchidism, without considering the possibility of anorchia in dogs (Amann & Veeramachaneni, 2006; Switonski et al., 2012). Previous studies have described diagnostic methodology for gonad developmental disorders in dogs, but to the best of the authors’ knowledge, this is the first study to provide a complete and appropriate diagnosis of complete anorchia in dogs. The absence of both gonads is often related to other sexual abnormalities, such as hypospadias (Rezaei et al., 2016) and micropenis (Cassata et al., 2008). The association between testicular dysgenesis and hypospadias is known as testicular dysgenesis syndrome (Hatipoğlu & Kurtoğlu, 2013). To implement an accurate diagnostic procedure, the authors have referred to relevant literature from human medicine (Toppari et al., 2010; Morgentaler et al., 2016; Harrington & Palmert, 2012) to reach an accurate and correct diagnosis based on both clinical criteria and instrumental tests (Aversa & Morgentaler, 2015).

## 2 AETIOLOGY

The causes that may lead to the development of an anorchid dog are still unknown, and too few studies have been carried out on this species to be able to define an obvious aetiology (Lyle, 2007). Based on the physiology of the formation and development of the genital organs, it is possible to imagine which points should be investigated in order to find a real correlation between cause and effect. Normal sexual differentiation occurs in three distinct stages: chromosomal (genetic) sex formation, gonadal sex development, and phenotypic sex development. In relation to the chromosomal sex, all embryos in the primary stages are sexually undifferentiated; they have bipotential genital ridges, Wolff and Muller's ducts, a urogenital sinus, a genital tubercle, and genital swellings. The genital chromosome constitution determines gonadal differentiation: The presence of a Y chromosome will lead to the differentiation of the genital ridge into a testis; conversely, the presence of an X chromosome will determine the development of the ovary. The *SRY* (sex‐determining region Y) gene present on the paternal chromosome is indispensable for the activation of genes leading to the development of a testis and for the suppression of those leading to the development of an ovary. The exact mechanism of operation of the *SRY* gene is still unknown (Lyle, 2007). However, functional testicular tissue must be present in early foetal life for Sertoli cells to secrete anti‐Müllerian hormone (AMH), leading to Müllerian duct regression, and for Leydig cells to produce androgens to induce the differentiation of Wolffian ducts, the urogenital sinus, and external genitalia (Rey & Grinspon, 2011). Genetic causes and mechanical causes, such as spermatic vessel occlusion caused by torsion or trauma during testicular descent in isolated cases, have been proposed to explain gonadal vanishing or regression occurring after the critical period of foetal sex differentiation (Vinci et al., 2004). In human medicine, this pathology is found in one out of every 20,000 males (Bobrow & Gough, 1970). The literature proposes three different syndromes as aetiologies. The first is the 46XY gonadal agenesis syndrome, which is characterised by a normal XY genotype and an ambiguous sexual phenotype, with abnormal external and internal genitalia. The cause is the lack of action of testicular development factors early in foetal life (Philibert et al., 2007; Petersen et al., 2008). The second syndrome is bilateral congenital anorchia, a condition characterised by a male XY genotype to which a male‐like phenotype corresponds, and all structures attached (located near) to the gonads are present except the testes. In this case, the lack of action of testicular development factors occurs late in pregnancy. The third possible syndrome is testicular regression syndrome, occurring during foetal life and involving atrophy and subsequent disappearance of an initially normal testis due to vascular problems (Bader et al., 2011). It is characterised by the presence of a rudimentary spermatic cord in the absence of macroscopically visible testicular tissue (Spires et al., 2000). These patients are genotypically and phenotypically male (Antic et al., 2011).

## 3 DIAGNOSIS

The diagnostic process must begin by gathering the patient's medical history, followed by a clinical visit with the patient, a general examination, and an examination of the genital system. An anorchid patient, with a low level of androgen hormones, shows a reluctance to move, ataxia, claudicated gait, painless hypotrophic hind limbs, and poor resistance to physical stress (Cicirelli et al., 2021). Obviously, the clinical signs to consider when diagnosing anorchia are lack of testicles and an empty or absent scrotum (Lyle, 2007). A particular physical examination is essential to recognise any cases of cryptorchidism and the presence of testicles in the inguinal ring of the dog (Romagnoli, 1991). When the testicle is not palpable, the problem arises in the differential diagnosis between cryptorchidism and congenital anorchia. Some authors have found anorchid dogs with concomitant andrological clinical signs, such as hypospadias (Rezaei et al., 2016) or micropenis (Lyle, 2007). In medium‐sized breeds, a penis with a length of 2.8 cm can be classified as a micropenis (Hatipoğlu & Kurtoğlu, 2013). The diagnosis of anorchia can also be made using ultrasound. The use of diagnostic imaging is useful for establishing the presence of gonads inside the patient's abdomen in the case of cryptorchidism (Khan et al., 2018). The use of ultrasound also serves to identify any problems such as prostatic hypotrophy, which is characterised by a lack of testosterone and therefore testicular agenesis (Cicirelli et al., 2021). Ultrasound is a good diagnostic method for cryptorchidism, with a sensitivity of 96.6% for the detection of abdominal testes and 100% for the detection of inguinal testes (Khan et al., 2018). For this reason, it could be used as an excellent method to exclude the possibility of cryptorchidism in diagnosing anorchid subjects. As indicated in the relevant literature, the anorchid patient should be subjected to ultrasound and then to computed tomography (CT) or magnetic resonance imaging (MRI) of the genital system, which must confirm the imaging diagnosis of testicular agenesis. MRI has a sensitivity of 85–86% and a specificity of 79–87%, but it is an expensive technique and requires sedation while not exposing the patient to ionising radiation (Ritzen, 2008). In the present review, the diagnostic process involved hormonal and genetic investigations subsequent to diagnostic imaging. Examination of the karyotype is necessary to determine the chromosomal sex of the child and exclude syndromic forms, which is especially indicated in the presence of associated anomalies. In fact, the anorchid patient must have an XY karyotype and must therefore be genetically male (King et al., 2006). To diagnose anorchia, it is useful to measure the patient's luteinizing hormone (LH), the pituitary gonadotropin that induces the production of testosterone in male Leydig cells. In fact, in an animal without gonads, since there is no feedback for the hypothalamus, gonadotropins are constantly released. This condition is detectable in dogs with a constant LH secretion of > 1 ng/mL (Spires et al., 2000). An AMH test was performed on the dog's blood as a diagnostic tool to determine the presence of functional canine gonadal tissue (Themmen et al., 2016), and the extremely low levels confirmed the absence of functioning testicular tissue. This hormone has several diagnostic uses in males, such as distinguishing cases of anorchia from cases in which the testicles fail to descend into the scrotum by the age of six months (Bay et al., 2011) and are therefore located either in the abdomen or in the inguinal canal. In this regard, the evaluation of AMH is useful when diagnosing animals that have no testicles in the scrotal pouch. AMH is also useful for distinguishing cryptorchids and anorchids from previously castrated patients (Themmen et al., 2016; Bay et al., 2011). The anorchid patient had very low testosteronemia (< 80 ng∖dL) (England et al., 1989). In addition, after a testosterone stimulation test was performed by injecting human chorionic gonadotropin (hCG) (IM 250 IU), the value of tesosteronemia 1 h after the stimulation test remained unchanged (Cicirelli et al., 2021; England et al., 1989). In human medicine, in the absence of a testosterone response to the hCG test, the diagnosis of bilateral congenital anorchia is made, and further investigation using imaging methods and surgical exploration in search of the testicles is unnecessary. hCG has a negative predictive value of 100% and a positive predictive value of 89%; this condition is almost constantly associated with micropenis and marked hypoplasia of the scrotum (Abacı et al., 2013). Surgical exploration or laparoscopy has a sensitivity and specificity close to 100% and generally makes it feasible to evaluate the possibility of repositioning the testicle to the scrotum and assessing the most suitable surgical approach (Kolon et al., 2014; Comploj & Pycha, 2012). The summary of the procedure for the diagnosis of anorchia is described in Table 1.

**TABLE 1:** Diagnostic procedure in case of suspected anorchia

**Cryptorchidism: Diagnostic Procedure** General and physical examinations Ultrasound and Echocolour Doppler of the inguino‐scrotal region CT or MRI of the abdominal pelvis LH dosage, testosterone, and anti‐Müllerian hormone hCG test → differential diagnosis between cryptorchidism, testicular ectopia, and congenital anorchia (no testosterone response to the hCG test → bilateral congenital anorchia)

## 4 THERAPY

As this problem presents little relevant literature, there is no medical treatment available. Symptoms of androgen deficiency include infertility, shrinkage of the penis and prostate, low muscle mass, and osteoporosis (Bremner, 2003). Supportive integrative therapy is very important in animals with muscle atrophy and bone deformation (Wierman et al., 2014). In addition, therapy for concomitant diseases is recommended for anorchid dogs (Cicirelli et al., 2021). The treatment of anorchia must be aimed at solving related problems. For example, Rezaei et al. (2016) treated subjects surgically to solve the problems of hypospadias in anorchid subjects. Cicirelli et al. (2021) suggested that the medical resolution of thyroid hormonal problems was probably related to the hormonal problems of an anorchid patient. Single‐ or multiport laparoscopic‐assisted approaches can be used for the diagnosis of cryptorchidism or anorchia in dogs and eventually for cryptorchidectomy (Miller et al., 2004; Cicirelli et al., 2021). Notable advantages over the traditional laparotomy method include minimal invasiveness, good visibility of the abdominal structures, no intraoperative complications, and no major postoperative complications (Miller et al., 2004). Some authors have described heterologous testicular transplantation in anorchid dogs. These studies have mostly focused on transplant‐related immunosuppressive therapy. Because of the similarity in vessel diameter with humans, dogs were chosen as subjects for the experiments (Barten et al., 1997). However, this study demonstrated that heterologous transplantation is possible if associated with immunosuppressive therapy (Barten et al., 1997).

To conclude, this review may provide the foundation for further investigations to provide an update on canine anorchia diagnosis that can be expanded in future studies.

## AUTHOR CONTRIBUTIONS

All authors collaborated in the same way for the drafting of this work.

## CONFLICT OF INTEREST

The authors state that they have no conflicts of interest.

### PEER REVIEW

The peer review history for this article is available at https://publons.com/publon/10.1002/vms3.1033

## Data Availability

The data presented in this study are available on request from the corresponding author.

## References

[vms31033-bib-0001] Abacı, A. , Çatlı, G. , Anık, A. , & Böber, E. (2013). Epidemiology, classification and management of undescended testes: Does medication have value in its treatment. Journal of Clinical Research in Pediatric Endocrinology, 5(2), 65–72. 23748056 10.4274/Jcrpe.883 PMC3701924

[vms31033-bib-0002] Adam, M. P. , Fechner, P. Y. , Ramsdell, L. A. , Badaru, A. , Grady, R. E. , Pagon, R. A. , McCauley, E. , Cheng, E. Y. , Parisi, M. A. , & Shnorhavorian, M. (2012). Ambiguous genitalia: what prenatal genetic testing is practical? American Journal of Medical Genetics, 158A, 1337–1343. 22581420 10.1002/ajmg.a.35338

[vms31033-bib-0003] Amann, R. P. , & Veeramachaneni, D. N. R. (2006). Testicular dysgenesis in animals: Cryptorchidism. Animal Reproduction, 3, 108–120.

[vms31033-bib-0004] Antic, T. , Hyjek, E. M. , & Taxy, J. B. (2011). The vanishing testis: A histomorphologic and clinical assessment. American Journal of Clinical Pathology, 136(6), 872–880. 22095372 10.1309/AJCPWPSJSK58RFUI

[vms31033-bib-0005] Aversa, A. , & Morgentaler, A. (2015). The practical management of testosterone deficiency in men. Nature Reviews Urology, 12, 641–650. 26458755 10.1038/nrurol.2015.238

[vms31033-bib-0006] Bader, M. I. , Peeraully, R. , Baath, M. , McPartland, J. , & Baillie, C. (2011). The testicular regression syndrome—Do remnant require routine excision. Journal of Pediatric Surgery, 46, 384–386. 21292092 10.1016/j.jpedsurg.2010.11.018

[vms31033-bib-0007] Barten, E. , Garybian, H. , Klopper, P. , & Newling, D. W. (1997). Homologous testis transplantation in dogs. Transplant International, 10, 362–368. 9287401 10.1007/s001470050071

[vms31033-bib-0008] Bay, K. , Main, K. M. , Toppari, J. , & Skakkebæk, N. E. (2011). Testicular descent: INSL3, testosterone, genes and the intrauterine milieu. Nature Reviews Urology, 8, 187–96. 21403659 10.1038/nrurol.2011.23

[vms31033-bib-0009] Bobrow, M. , & Gough, M. H. (1970). Bilateral absence of testes. Lancet, 295, 366. 10.1016/s0140-6736(70)90753-1 4189631

[vms31033-bib-0010] Bremner, W. J. (2003). Androgens in health and disease. Humana Press. pp. 365–379. ISBN 978‐1‐58829‐029‐8. Retrieved 11 June 2012.

[vms31033-bib-0011] Cassata, R. , Iannuzzi, A. , Parma, P. , De Lorenzi, L. , Peretti, V. , Perucatti, A. , Iannuzzi, L. , & Di Meo, G. P. (2008). Clinical, cytogenetic and molecular evaluation in a dog with bilateral cryptorchidism and hypospadias. Cytogenetic and Genome Research, 120, 140–143. 18467838 10.1159/000118753

[vms31033-bib-0012] Cicirelli, V. , Aiudi, G. G. , Carbonara, S. , Caira, M. , & Lacalandra, G. M. (2021). Case of anorchia in a mixed‐breed dog. Topics in Companion Animal Medicine, 45, 100554. 34192562 10.1016/j.tcam.2021.100554

[vms31033-bib-0013] Cicirelli, V. , Debidda, P. , Maggio, N. , Caira, M. , Lacalandra, G. M. , & Aiudi, G. G. (2021). Ultrasound‐guided funicular block: Ropivacaine injection into the tissue around the spermatic cord to improve analgesia during orchiectomy in dogs. Animals, 11(5), 1275. 33925210 10.3390/ani11051275 PMC8146739

[vms31033-bib-0014] Comploj, E. , & Pycha, A. (2012). Diagnosis and management of cryptorchidism. European Urology Supplements, 11, 2–9.

[vms31033-bib-0015] England, G. C. , Allen, W. E. , & Porter, D. J. (1989). Evaluation of the testosterone response to hCG and the identification of a presumed anorchid dog. Journal of Small Animal Practice, 30, 441–443.

[vms31033-bib-0016] Harrington, J. , & Palmert, M. R. (2012). Clinical review: Distinguishing constitutional delay of growth and puberty from isolated hypogonadotropic hypogonadism: critical appraisal of available diagnostic tests. Journal of Clinical Endocrinology and Metabolism, 97, 3056–3067. 22723321 10.1210/jc.2012-1598

[vms31033-bib-0017] Hatipoğlu, N. , & Kurtoğlu, S. (2013). Micropenis: Etiology, diagnosis and treatment approaches. Journal of Clinical Research in Pediatric Endocrinology, 5(4), 217–223. 24379029 10.4274/Jcrpe.1135 PMC3890219

[vms31033-bib-0018] Khan, F. A. , Gartley, C. J. , & Khanam, A. (2018). Canine cryptorchidism: An update. Reproduction in Domestic Animals = Zuchthygiene, 53(6), 1263–1270. 29956390 10.1111/rda.13231

[vms31033-bib-0019] King, R. C. , Stansfield, W. D. , & Mulligan, P. K. (2006). A dictionary of genetics (7th ed.). Oxford University Press.

[vms31033-bib-0020] Kolon, T. F. , Herndon, C. D. , Baker, L. A. , Baskin, L. S. , Baxter, C. G. , Cheng, E. Y. , Diaz, M. , Lee, P. A. , Seashore, C. J. , Tasian, G. E. , & Barthold, J. S. (2014). Evaluation and treatment of cryptorchidism: AUA guideline. Journal of Urology, 192(2), 337–345. 24857650 10.1016/j.juro.2014.05.005

[vms31033-bib-0021] Lyle, S. K. (2007). Disorders of sexual development in the dog and cat. Theriogenology, 68(3), 338–343. 10.1016/j.theriogenology.2007.04.015. Epub May 4. 17482251

[vms31033-bib-0022] Lyle, S. K. (2007). Disorders of sexual development in the dog and cat. Theriogenology, 68, 338–343. 17482251 10.1016/j.theriogenology.2007.04.015

[vms31033-bib-0023] Miller, N. A. , Van Lue, S. J. , & Rawlings, C. A. (2004). Use of laparoscopic‐assisted cryptorchidectomy in dogs and cats. Journal of American Veterinary Medical Association, 224, 875–878. 10.2460/javma.2004.224.875 15070057

[vms31033-bib-0024] Morgentaler, A. , Zitzmann, M. , Traish, A. M. , Fox, A. W. , Jones, T. H. , Maggi, M. , Arver, S. , Aversa, A. , Chan, J. C. , Dobs, A. S. , Hackett, G. I. , Hellstrom, W. J. , Lim, P. , Lunenfeld, B. , Mskhalaya, G. , Schulman, C. C. , & Torres, L. O. (2016). Fundamental concepts regarding testosterone deficiency and treatment: International Expert Consensus Resolutions. Mayo Clinic Proceedings, 91, 881–896. 27313122 10.1016/j.mayocp.2016.04.007

[vms31033-bib-0025] Petersen, R. O. , Sesterhenn, I. A. , & Davis, C. J. (2008). Urologic pathology. Lippincott Williams & Wilkins.

[vms31033-bib-0026] Philibert, P. , Zenaty, D. , Audran, F. , Leger, J. , Acherman, J. , & Sultan, C. (2007). Mutational analysis of steroidogenic factor 1 (NR5a1) in 24 boys with bilateral anorchia: A French collaborative study. Human Reproduction, 22, 3255–3261. 17940071 10.1093/humrep/dem278 PMC2990861

[vms31033-bib-0027] Rey, R. A. , & Grinspon, R. P. (2011). Normal male sexual differentiation and aetiology of disorders of sex development. Best Practice & Research Clinical Endocrinology & Metabolism, 25, 221–238. 21397195 10.1016/j.beem.2010.08.013

[vms31033-bib-0028] Rezaei, M. , Azizi, S. , Akhtardanesh, B. , Azari, O. , Hassibi, H. , & Bojd, T. (2016). Hypospadias and testicular agenesis in two German shepherd puppies. Iranian Journal of Veterinary Surgery, 11(1), 51–55.

[vms31033-bib-0029] Ritzen, E. (2008). Undescended testis: A consensus on management. European Journal of Endocrinology, 159, S87–S90. 18728121 10.1530/EJE-08-0181

[vms31033-bib-0030] Romagnoli, S. E (1991). Canine cryptorchidism. Veterinary clinics of North America. Small Animal Practice, 21, 533–544. 1677504 10.1016/s0195-5616(91)50059-0

[vms31033-bib-0031] Spires, S. E. , Woolums, C. , Pulito, A. R. , & Spires, S. M. (2000). Testicular regression syndrome. A clinical and pathologic study of 11 cases. Archives of Pathology & Laboratory Medicine, 124, 694–698. 10782149 10.5858/2000-124-0694-TRS

[vms31033-bib-0032] Switonski, M. , Payan‐Carreira, R. , Bartz, M. , Nowacka‐Woszuk, J. , Szczerbal, I. , Colaço, B. , Pires, M. A. , Ochota, M. , & Nizanski, W. (2012). Hypospadias in a male (78,XY; SRY‐positive) dog and sex reversal female (78,XX; SRY‐Negative) dogs: Clinical, histological and genetic studies. Sexual Development, 6, 128–134. 21893969 10.1159/000330921

[vms31033-bib-0033] Themmen, A. P. L. , Kalra, B. , Visser, J. A. , Kumar, A. , Savjani, G. , Gier, J. , & Jacques, S. (2016). The use of anti‐Müllerian hormone as diagnostic for gonadectomy status in dogs. Theriogenology, 86(6), 1467–1474. 27291082 10.1016/j.theriogenology.2016.05.004

[vms31033-bib-0034] Thorup, J. , McLachlan, R. , Cortes, D. , Nation, T. R. , Balic, A. , Southwell, B. R. , & Hutson, J. M. (2010). What is new in cryptorchidism and hypospadias—A critical review on the testicular dysgenesis hypothesis. Journal of Pediatric Surgery, 45(10), 2074–2086. 20920735 10.1016/j.jpedsurg.2010.07.030

[vms31033-bib-0035] Toppari, J. , Virtanen, H. E. , Main, K. M. , & Skakkebaek, N. E. (2010). Cryptorchidism and hypospadias as a sign of testicular dysgenesis syndrome (TDS): Environmental connection. Birth Defects Research (Part A), 88, 910–919. 20865786 10.1002/bdra.20707

[vms31033-bib-0036] Vinci, G. , Anjot, M. N. , Trivin, C. , Lottmann, H. , Brauner, R. , & McElreavey, K. (2004). An analysis of the genetic factors involved in testicular descent in a cohort of 14 male patients with anorchia. Journal of Clinical Endocrinology Metabolism, 89, 6282–6285. 15579790 10.1210/jc.2004-0891

[vms31033-bib-0037] Wierman, M. E. , Arlt, W. , Basson, R. , Davis, S. R. , Miller, K. K. , Murad, M. H. , Rosner, W. , & Santoro, N. (2014). Androgen therapy in women: A reappraisal: An Endocrine Society clinical practice guideline. The Journal of Clinical Endocrinology and Metabolism, 99(10), 3489–510. 25279570 10.1210/jc.2014-2260

